# Tannic acid and silicate-functionalized polyvinyl alcohol–hyaluronic acid hydrogel for infected diabetic wound healing

**DOI:** 10.1093/rb/rbae053

**Published:** 2024-05-13

**Authors:** Zhentian Diao, Longkang Li, Huan Zhou, Lei Yang

**Affiliations:** School of Materials Science and Engineering, Hebei University of Technology, Tianjin 300131, China; School of Materials Science and Engineering, Hebei University of Technology, Tianjin 300131, China; Center for Health Science and Engineering, Hebei Key Laboratory of Biomaterials and Smart Theranostics, School of Health Sciences and Biomedical Engineering, Hebei University of Technology, Tianjin 300131, China; Center for Health Science and Engineering, Hebei Key Laboratory of Biomaterials and Smart Theranostics, School of Health Sciences and Biomedical Engineering, Hebei University of Technology, Tianjin 300131, China

**Keywords:** diabetic wound healing, ROS overexpression, combating infection, stimulate angiogenesis

## Abstract

Healing of chronic diabetic wounds is challenging due to complications of severe inflammatory microenvironment, bacterial infection and poor vascular formation. Herein, a novel injectable polyvinyl alcohol–hyaluronic acid-based composite hydrogel was developed, with tannic acid (TA) and silicate functionalization to fabricate an ‘all-in-one’ hydrogel PTKH. On one hand, after being locally injected into the wound site, the hydrogel underwent a gradual sol–gel transition *in situ*, forming an adhesive and protective dressing for the wound. Manipulations of rheological characteristics, mechanical properties and swelling ability of PTKH could be performed via regulating TA and silicate content in hydrogel. On the other hand, PTKH was capable of eliminating reactive oxygen species overexpression, combating infection and generating a cell-favored microenvironment for wound healing acceleration *in vitro*. Subsequent animal studies demonstrated that PTKH could greatly stimulate angiogenesis and epithelization, accompanied with inflammation and infection risk reduction. Therefore, in consideration of its impressive *in vitro* and *in vivo* outcomes, this ‘all-in-one’ multifunctional hydrogel may hold promise for chronic diabetic wound treatment.

## Graphical abstract

**Figure:**
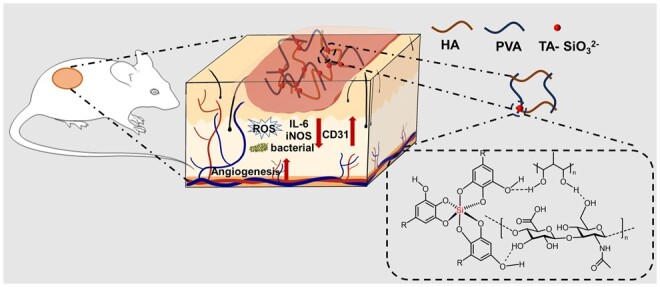

## Introduction

To date, there are nearly 500 million diabetes patients worldwide [1–3], among whom 19–34% are prone to chronic diabetic wounds [4]. The complications of diabetic wounds are attributed to the incidence of local ischemia and tissue necrosis, as well as limited resistance to infections. These disorders are mainly caused by high sugar microenvironment-induced poor vascular formation and severe inflammatory microenvironment accompanied with large amounts of reactive oxygen species (ROS) generation *in situ* [5–7]. Therefore, in clinical practice, it is essential to promote angiogenesis and control inflammation in wounds [8]. Such medical needs thereby accelerate evolution of functional dressing biomaterials targeting angiogenesis and inflammation, in the form of sponges, hydrogels, foams, hydrocolloids, fiber membranes and so forth [9–11].

In the progression of chronic diabetic wounds, the injured site often begins with a serious inflammatory reaction [12], in which the M1 macrophage phenotype is usually predominant. The transition to the M2 type is greatly inhibited due to the increase in pro-inflammatory cytokines induced by the hyperglycemic environment [13]. Moreover, prolonged inflammation is observed to reduce the generation of proangiogenic factors, as well as increase risks of pathological scars and infection. Therefore, it is essential to control inflammation *in situ* using functional biomaterials [14–16].

Hydrogels, because of their ease of administration to wounds and biocompatibility, are acknowledged as the most representative dressing biomaterials in chronic diabetic wound treatments [17, 18]. On one hand, the physical characteristics of hydrogels enable them to provide a moist and oxygen-permeable local environment that accelerates wound healing and acts as a swellable platform for absorbing wound exudates. For example, researchers reported a freezing hydrogel matrix characterized by interpenetrated porous structures, showing outstanding breathability and great capability in wound exudate management [19]. On the other hand, numerous active pharmaceutical ingredients can be loaded into a hydrogel matrix to regulate the microenvironment for angiogenesis and reduce inflammation and infection levels [20–22]. In addition to these therapeutic properties, the hydrogel can be further endowed with tissue-adhesive and hemostatic abilities to make it more applicable in wound treatment, aimed to provide a reliable platform to induce wound healing, halt bleeding, control wound exudate, and act as a barrier defending bacterial invasion [23, 24].

In the literature, tannic acid (TA) [25, 26] and silicate [27, 28] were both adopted in functionalizing hydrogel for inflammation and angiogenesis regulation. TA is a natural polyphenol with a large number of catechol or pyrogallol groups, which enables it showing efficient ROS scavenging, hemostatic and antibacterial abilities. In addition, the presence of TA in hydrogel can facilitate its adhesion to tissues by forming strong noncovalent bonds. In a work done by Yao et al. [15], cyclic freeze–thaw-treated polyvinyl alcohol (PVA)–chitosan hydrogel matrix was soaked into TA solution to generate an adhesive and hydrogen bond-reinforced antibacterial composite. On the other hand, silicate is an emerging anion in vascularization enhancement, capable of recruiting endothelial cells from peripheral tissues and enhancing the expression of specific growth factors to accelerate vascular regeneration [29, 30]. It also accelerates the recruitment of fibroblasts around the wound in terms of extracellular matrix (ECM) and collagen fiber deposition [31]. Hyaluronic acid (HA), a naturally occurring polysaccharide produced by fibroblasts during the proliferation of fibroblasts during the wound repair phase, plays an important role in wound tissue regeneration and angiogenesis, as well as reduces inflammation and enhances collagen deposition at the wound site [32]. Given these characteristics, the integration of TA and silicate to generate an appropriate microenvironment to suppress inflammation and accelerate angiogenesis could be adopted in hydrogel design, as an effort to address the above-described challenges of chronic diabetic wounds.

Inspired by the above descriptions, in our current work, we developed a PVA and HA-based composite hydrogel empowered with both TA and silicate for diabetic-infected wound dressing. It was found due to the crosslink network formation among TA, silicate, PVA and HA, the fluidic mixture could be gelatinized *in situ* to form an ‘all-in-one’ protective barrier, providing convenience in wound care. Nevertheless, this ‘all-in-one’ hydrogel revealed benefits in regulating inflammation, suppressing wound infection, eliminating bleeding and enhancing angiogenesis, overall demonstrating ultimately diabetic wound healing acceleration.

## Materials and methods

### Materials

PVA (1799) and 2,2-diphenyl-1-picrylhydrazyl were provided by Shanghai Aladdin Industrial Co., Ltd (Shanghai, China). K_2_SiO_3_ was purchased from Tianjin Guangfu Industrial Co., Ltd (Tianjin, China). TA and HA (*M*_w_ 400–800 kDa) were provided by Shanghai McLean Co., Ltd (Shanghai, China). Live/dead fluorescence kit (Calcein-AM/PI), CCK8, 2,7-dichlorodihydrofluorescein diacetate (DCFH-DA) and streptozocin were purchased from Shanghai Beyotime Biochemical Technology Co., Ltd (Shanghai, China).

### Hydrogel preparation

For the preparation of the TA and silicate-integrated composite hydrogel, 0.1 g of TA was dissolved in 7.5 ml of de-ionized water. Subsequently, 1 g of PVA was added and mixed well, followed by 2 h of stirring at 95°C. On the other hand, a silicate solution was simultaneously prepared by adding 0.1 g of K_2_SiO_3_ to 2.5 ml of HA solution (4 mg/ml). The ‘all-in-one’ hydrogel, referred to as PTKH1 was then generated by mixing both solutions together, along with a sol–gel transition. Following the same procedure, PTKH2 and PTKH3 were prepared with the amount of TA and K_2_SiO_3_ simultaneously increased to 0.15 and 0.2 g. On the other hand, a PVA–HA hydrogel (PH) in the absence of TA and silicate was also prepared for comparison. The formula of hydrogel samples are summarized in Table 1. Regarding this formulated hydrogel lacking self-gelation ability, the liquid was poured into a mold and proceeded with three cycles of freeze–thaw treatment to generate a solid hydrogel matrix.

**Table 1.** PH and PTKH hydrogel material ratios

	DI (ml)	PVA (g)	HA (mg)	TA (g)	K_2_SiO_3_ (g)
PH	10	1	10		
PTKH1	10	1	10	0.1	0.1
PTKH2	10	1	10	0.15	0.15
PTKH3	10	1	10	0.2	0.2

### Rheological tests

A rheometer was used to assess the rheological characteristics of PTKH1, PTKH2 and PTKH3 hydrogels, utilizing a plate-to-plate configuration. The parameters were set at 1% strain for angular velocities ranging from 0.1 to 100 at 25°C.

### Tensile tests

PTKH hydrogel samples (50 × 5 × 3 mm in length, width and thickness) were subjected to tensile testing (100 mm/min) by a tensile testing machine and the tensile strength and elongation at break of the resulting samples were calculated.

### Swelling test

The PTKH samples were placed in deionized water (37°C). The PTKH samples were then weighed and recorded. At a specific time, the PTKH sample was removed from the surface after excess water was removed and weighed to record. The water absorption of the test sample was calculated using the following equation:

$$\mathrm{Degree}\;\mathrm{of}\;\mathrm{swelling}=\frac{Wi-Wo}{Wo}\times 100\%$$

*W*_i_: sample mass after swelling

*W*_o_: initial sample mass before swelling

### Radical scavenging activity

A DPPH assay was adopted to examine the radical scavenging activity of PTKH hydrogels. Briefly, DPPH was dissolved in methanol to make a stocking solution (100 μM), followed by immersing *in situ* crosslinked hydrogels into DPPH (50 mg/ml). After 30-min proceeding, readings were taken at 517 nm using an enzyme marker. The free radical scavenging effect of different PTKH hydrogels was calculated using the following equation:

$$\mathrm{DPPH}\left(\mathrm{cleaning}\;\mathrm{rate}\%\right)=\frac{Ai-Af}{Af}\times 100\%$$

*A*_i_: OD of initial DPPH solution at 517 nm

*A*_f_: OD of DPPH after reaction with hydrogel at 517 nm

### Hemolysis and coagulation

In chronic diabetic wound care, the incidence of bleeding is common due to the loss of skin sensitivity to impairment and disorder of tissue regeneration. Therefore, regarding the direct interaction between hydrogel and blood, satisfactory hemostatic properties are required for the biomedical practice of PTKH [33]. In the hemolysis study, 100 mg of hydrogel was co-incubated with 2 ml red blood cell suspension at 37°C for 1 h (50 mg/ml). After incubation, it was centrifuged at 1600 rpm for OD test at 540 nm. Meanwhile, the coagulation test was carried out using the blood clotting index (BCI) method, in which 0.2 g of 37°C pre-warmed PTKH hydrogel was exposed to a freshly prepared mixture of 50 μl of sheep anticoagulated blood. After 10 min incubation, 20 ml of deionized water was added, along with OD value tested at 540 nm.

$$\begin{array}{c} \mathrm{Hemolysis}\;\left(\%\right)=\frac{Ai-As}{At-As}\times 100\%\\ B\mathrm{CI}\left(\%\right)=\frac{Ai}{At}\times 100\% \end{array}$$

*A*_i_: OD of hydrogel group at 540 nm

*A*s: OD of the saline-treated group at 540 nm (negative control)

*A*t: OD of positive control at 540 nm

### *In vitro* antibacterial test

In this work, we chose to use gram-positive *Staphylococcus aureus* (*S.aureus*) and gram-negative *Escherichia coli* (*E.coli*) served as testing bacteria. A suspension of 1 × 10^5^ CFU/ml of Luria–Bertani bacteria was co-cultured with PTKH and PH hydrogel for 24 h. Bacterial survival was assessed by the smear plate counting method.

### Cell proliferation test

We used HUVECs and L929 cells to evaluate the cytocompatibility, ROS scavenging and angiogenic activity of PTKH hydrogels.

Initially, the sterilized hydrogel was incubated in centrifuge tubes containing Dulbecco’s modified Eagle’s medium for 24 h (50 mg/ml) to prepare hydrogel extract. Subsequently, the extract was filtered through a 0.22 μm membrane, and the filtered extract was added to 10 vol% fetal bovine serum (FBS) and 1 vol% penicillin–streptomycin solution (P/S). All medium used in the experiment were purchased from Gibco Life Technologies.

The proliferation behavior of L929 and HUVECs in a hydrogel extract-based medium was investigated by seeding 4000 cells to 100 μl culture medium in a 96-well plate. After incubation for 24 and 96 h, CCK-8 was added following the kit protocol. After incubation, OD at 450 nm was detected and cell viability was calculated using the following equation:

$$\mathrm{Cell}\;\mathrm{viability}\;\left(\%\right)=\frac{\left(\mathrm{ODi}-\mathrm{ODs}\right)}{\left(\mathrm{ODo}-\mathrm{ODs}\right)}\times 100\%$$

OD_i_: OD value of hydrogel extract

OD_o_: OD value of the control group

OD_s_: OD value of background

The viability of HUVECs and L929 cells in the extracts was assessed using live/dead staining and photographed using fluorescence microscopy.

### Intracellular ROS scavenging

We used DCFH-DA to characterize the intracellular antioxidant capacity of PTKH extracts. After L929 was inoculated in hydrogel extract for 24 h, Rosup reagent was added to generate intracellular ROS. Following another 7-h incubation, a dichlorodihydrofluorescein (DCFH-DA) cell fluorescence probe was added following the kit protocol. In addition, cells cultured with or without Rosup reagent were set as positive and negative groups, respectively. In addition, the positive group was the group of cells containing only Rosup reagent, while the negative group was the group of cells without Rosup. Finally, photographs were taken with a fluorescence microscope and processed for fluorescence intensity using Image J.

### Cell migration assay

We grew HUVECs in 12-well plates. When cells formed a monolayer, cell-free scratches were made with a 200-μl pipette tip. The plates were then washed repeatedly with PBS to remove free cells, and the original medium was replaced with a medium containing 2% FBS or corresponding extracts. The status of HUVECs at 0, 12 and 24 h after the formation of scratches was observed by light microscopy.

### Transwell assay

We seeded HUVECs into the upper chamber (5 × 10^4^ cells/well) and added hydrogel extract to the lower chamber. After 24 h of incubation, cells were treated with 4% paraformaldehyde and 0.1% violet crystals.

### Tube formation assay

HUVECs were seeded at a density of 2 × 10^4^ per well in Matrigel supplemented 96-well plates and cultured for 6 h, and then observed and photographed by light microscopy and analyzed by Image J.

### *In vivo* test

Male BALB/c mice (∼20 g) were injected intraperitoneally with streptozotocin 120 mg/kg for 3 days, and blood glucose levels were measured using the tail-clamp method for 3 days, and blood glucose levels exceeding 11.1 mmol/l for three consecutive days were considered successful modeled [34].

The successfully modeled mice were randomly divided into five groups. A total loss skin wound of 10 mm diameter was created on the back of the animal. The untreated group was the control group. The PH, PTKH1, PTKH2 and PTKH3 groups were treated with the corresponding hydrogel. On the first day after surgery, *S.aureus* (1 × 10^6^ CFU/ml) was used to induce wound infection in animals. After 3 days, the infection level of the wound was evaluated via 1 ml of physiological saline rinsing followed by a colonies counting test in a petri dish. Wounds were photographed at specific time points and analyzed using Image J processing. After 15 days, the skin tissue of the wound site was fixed with 4% paraformaldehyde. The collected tissue samples were then processed, stained with H&E and Masson, and immunohistochemically stained with CD31, IL-6 and iNOS for analysis.

This experiment has been reviewed and approved by the animal experiment ethical inspection of Hebei University of Technology, approval number: HEBUTACUC2022001.

### Statistical analysis

Data were statistically analyzed by GraphPad Prism 8 software through. All quantitative data were expressed as mean ± standard deviation (SD) (*n* = 3). Statistically significant values are denoted as *P < 0.05, **P < 0.01, ***P < 0.001 and ****P < 0.0001, respectively.

## Results and discussion

### Fabrication and characterizations of PTKH hydrogels

Hydrogels have been extensively studied for chronic diabetic wound healing applications. Today, PVA is acknowledged as one of the most classical hydrogels in skin wound management [35]. However, PVA hydrogel possesses insufficient elasticity, unfavored stiffness and inadequate hydrophilic properties to restrict its potential in wound dressing, which in consequence drives the investigation of PVA-based composite hydrogel with additional polysaccharide integration [36, 37]. Among known polysaccharides, HA, a significant part of the skin ECM, attracts great attention in PVA hydrogel modification. The relevant PVA and HA-based composites are reported to yield moderate mechanical properties, good biodegradability and favored cell adhesion. However, the cyclic freeze–thaw method is the most applied method in generating PVA–HA hydrogel [38, 39], thus limiting its potential in irregular wound care as *in situ* gelatinized dressing. As an attempt to endow PVA–HA hydrogel the ability to form stable and adhesive dressing in skin wound, we designed a composite hydrogel functionalized with TA and silicate in current work.

**Figure 1.**
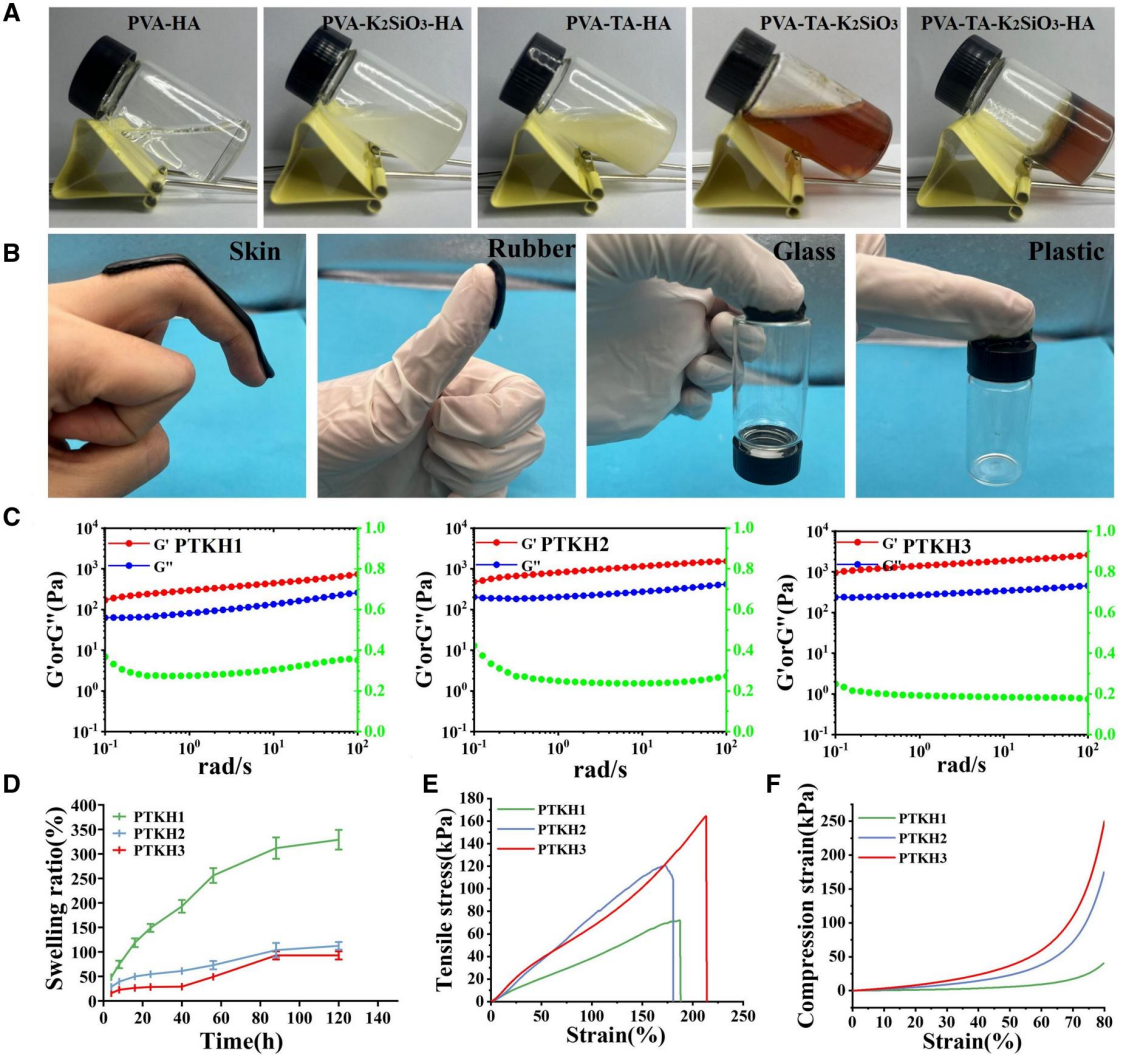
Characterization of PTKH hydrogels. (**A**) Gelation behavior difference of tested hydrogel formula; (**B**) Adhesion of PTKH hydrogel to different substances; (**C**) *G*′, *G*″ and *G*″/*G*′ ratio of PTKH hydrogel versus oscillation frequency; (**D**) Swelling behavior of PTKH hydrogels; (**E**) tensile test result; (**F**) compression test of PTKH hydrogel at 80% strain.

As shown in Figure 1A, mixture of PVA and HA prepared at room temperature appeared to be in viscous liquid state. When TA or silicate was added to the mixture alone, no transition of the liquid to gel was witnessed. However, once both TA and silicate were added to PVA–HA, a gel was formed quickly and did not flow along the walls of the test tube containing the mixture. It was also interesting to find out that when PVA was exposed to TA and silicate alone, the transition of the mixture from liquid to gel failed at room temperature. Therefore, the PVA and HA gelation could only be initiated when both TA and silicate were present, attributed to the formation of silicate–phenolic networks [40] and hydrogen bonds among PVA, HA and TA *in situ*. The absence of any component led to the destabilization of the crosslinked matrix and failure of gelatinization. Besides, the gel formation rate revealed a TA and silicate concentration-dependent behavior, in which the gelatinization time decreased from 25.4, 15.1 to 6.76 min (Supplementary Figure S1), in the order of PTKH1, PTKH2 and PTKH3. This observation could be explained by the gradual crosslinking density increase caused by increasing TA and silicate content in hydrogel formula, as well as the good injectability of PTKH (Supplementary Figure S2). In spite of the ability to adapt to wound shape, tested hybrid hydrogels showed adhesive ability (Supplementary Figure S3), attributed to the hydrogen bond formation between TA and substances (Figure 1B). Both characteristics make the hydrogel less prone to loss when acting as wound dressing.

The rheological properties of PTKH hydrogels were investigated (Figure 1C). PTKH3 hydrogel exhibited the highest storage modulus of 2.47 kPa (*G*′) among the three PTKH hydrogels. The storage modulus of 2.47 kPa (*G*′) of PTKH hydrogels decreased to 0.736 kPa (*G*′) with decreasing TA–silicate content (Figure 1C). This is due to the fact that the increase in TA and silicate content increases the crosslink density.

We investigated the swelling kinetics of PTKH hydrogels by weighing the water-swollen hydrogels at specific time points. The water absorption of PTKH hydrogel reached its maximum value when it was soaked for 120 h. The swelling ratios of PTKH1, PTKH2 and PTKH3 were 329%, 112% and 93%, respectively (Figure 1D). The existence of a substantial amount of hydrophilic groups in PVA and HA was acknowledged as the crucial factor in the hydrogel’s liquid absorption ability. While an increase in the TA and silicate content considerably restricted the swelling ability of hydrogels via increasing crosslinking density and leading to a dense structure.

We also examined the tensile and compressive characteristics of PTKH hydrogels. The stress–strain curves of PTKH hydrogels were tested as shown in Figure 1E. The results showed that elongation at break for PTKH1, PTKH2 and PTKH3 hydrogel groups were 188%, 180% and 211%, respectively. The corresponding PTKH hydrogel stresses ranged from 71 to 164 kPa, which was in agreement with the above-described rheological data. PTKH hydrogel was further evaluated by compression experiments (Figure 1F). The mechanical properties of PTKH show a gradual increase with the increase of its crosslinking degree with the increase of TA and K_2_SiO_3_ content in PTKH hydrogel. For diabetic wound treatment, a hydrogel with good tensile and compressive properties is expected to protect the wounds from further impairment and create a humid environment thereby promoting wound healing. Therefore, physical properties of PTKH hydrogels found in the testing demonstrated their potential as skin wound care materials.

### ROS scavenging activity

Chronic wounds often exhibit excessive expression of ROS in the wound site, leading to oxidative stress. In chronic wounds in diabetic patients, the unique high-sugar environment easily promotes bacterial growth, which in turn triggers ROS production in the wound site [41–43]. Excessive ROS can attack cells, leading to cell apoptosis, inducing inflammation, inhibiting blood vessel formation and impeding epithelialization, all of which prolong the healing time of a wound. Consequently, the creation of design antioxidant hydrogel to exert radical scavenging activity at the wound site, creating an oxidative stress suppressed microenvironment for cell proliferation and tissue regeneration.

**Figure 2.**
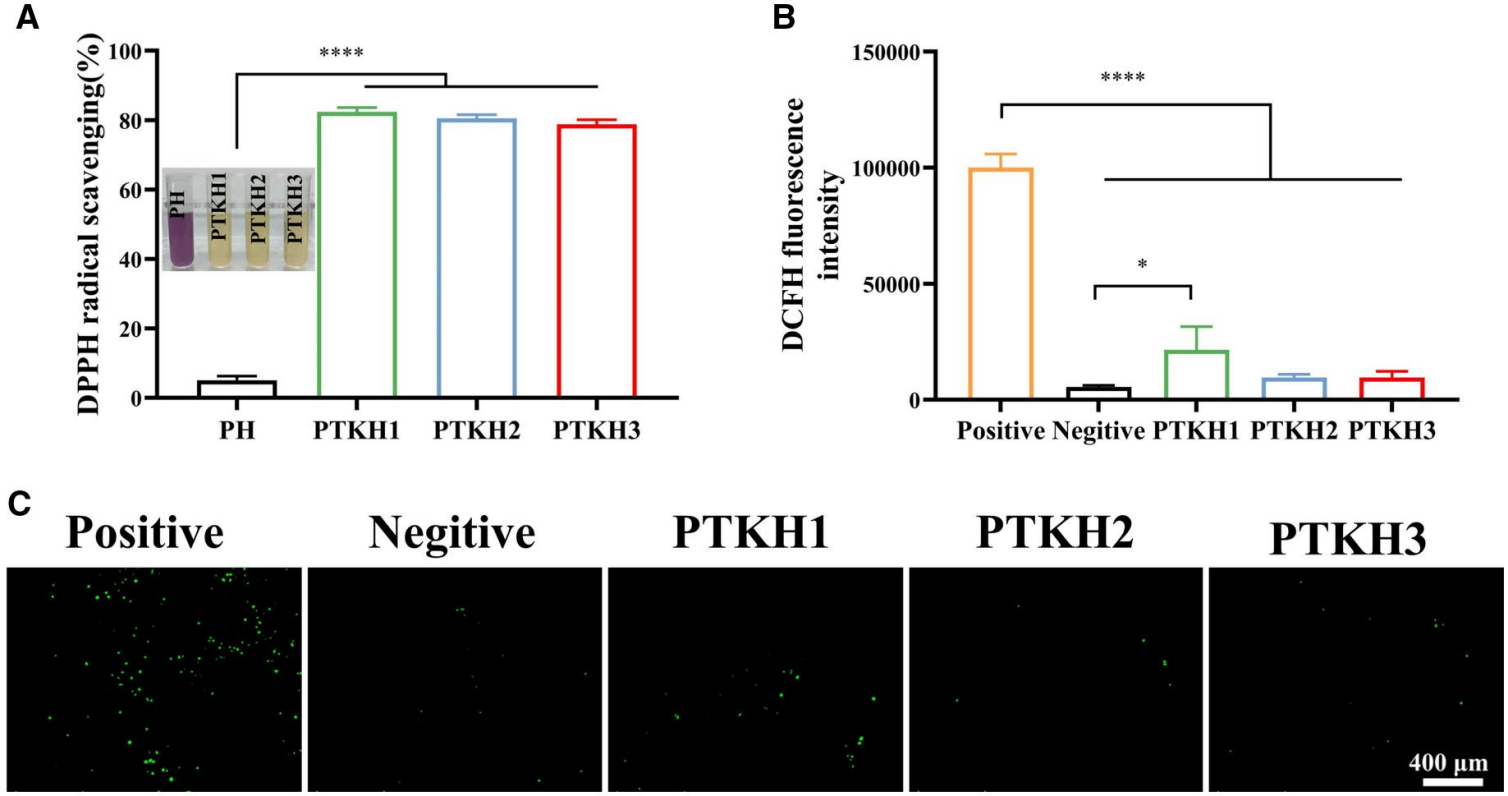
Antioxidant ability of PTKH hydrogel. (**A**) Relative free radical scavenging activity of hydrogels in DPPH solution; (**B**) intracellular ROS scavenging capacity; (**C**) ROS staining of L929 cells after exposed to hydrogel extract. *P < 0.05, ****P < 0.0001.

TA is a well-recognized antioxidant component that inhibits free radicals through electron transfer and scavenges overproduced ROS. Its presence in PTKH thereby was suggested to combat oxidative stress *in situ* [44]. We used both DPPH removal (Figure 2A) and the intracellular ROS scavenging model (Figure 2B and C) to demonstrate the antioxidation ability of PTKH hydrogels. On one hand, as shown in Figure 2A, three tested PTKH hydrogels all exhibited over 80% clearance efficiency of DPPH. On the other hand, compared to the positive group, the fluorescence brightness of L929 fibroblast cells was gradually dismissed in PTKH groups with increasing TA content in the hydrogel.

### Hemolysis and coagulation

**Figure 3.**
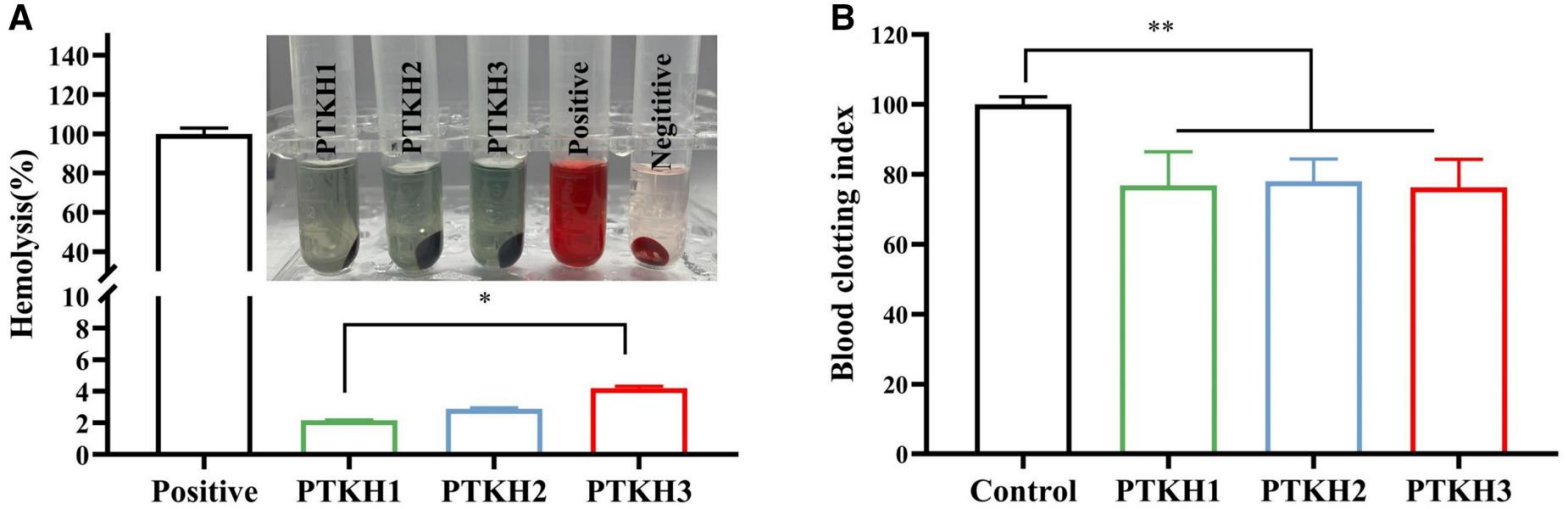
Hemolysis and coagulation of PTKH hydrogels. (**A**) Hemolysis rate of different test hydrogels; (**B**) coagulation index of different hydrogels. *P < 0.05, **P < 0.01.

The hemolysis test is a classical method to evaluate the hemocompatibility of the hydrogel in direct contact with blood. Figure 3A showed the hemolysis results of different tested groups, in which the PTKH hydrogel groups showed free TA release induced light dark green, the negative control was colorless, while the positive control (deionized water) was bright red in color. The hemolysis rate in the PTKH group was <4%, indicating that the PTKH hybrid hydrogel has good hemocompatibility. Besides, the hemostatic ability of PTKH hydrogels was also tested and compared with whole blood-clotting assay (Supplementary Figure S4). The control group did not show any clotting ability, while the PTKH hydrogel experimental groups exhibited visible blood clot formation on the hydrogel surface, with a BCI [33] value of ∼80% of the control (Figure 3B).

### *In vitro* antibacterial performance

**Figure 4.**
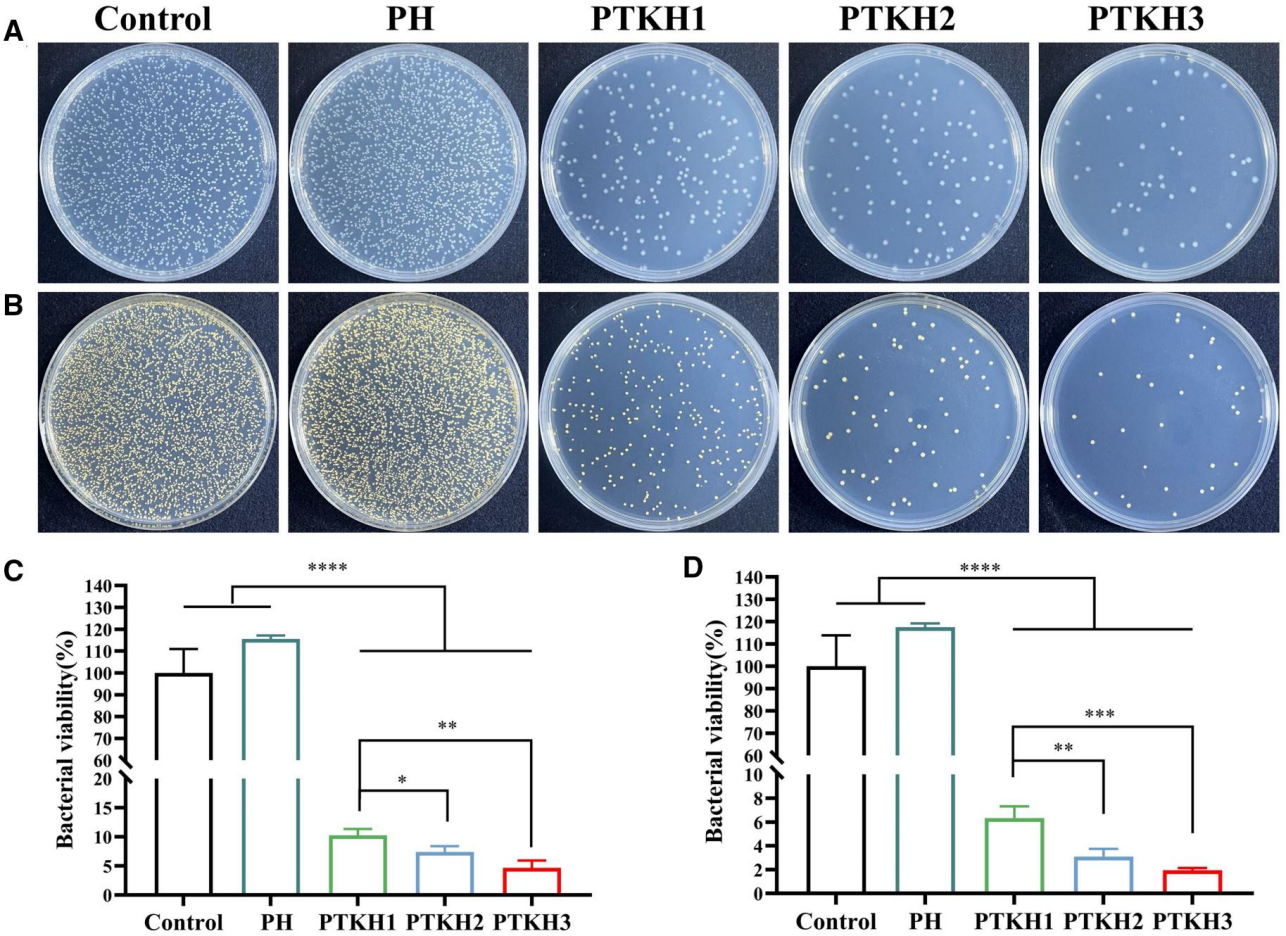
Antibacterial properties of PTKH hydrogels. (**A**) Photographs of *E.coli* proliferation after exposure to different hydrogels; (**B**) photographs of *S.aureus* proliferation after exposure to different hydrogels; (**C**) quantified analysis of *E.coli* colonies formation in tested groups; (**D**) quantified analysis of *S.aureus* colonies formation in tested groups. *P < 0.05, **P < 0.01, ***P < 0.001 ****P < 0.0001.

The diabetic wound is highlighted with a high-sugar environment and immune disorder, making it prone to bacterial infection. Therefore, wound dressing capable of bacterial inhibition is essential in medical practice [45, 46]. To demonstrate the antibacterial potential of PTKH, we chose *S.aureus* as a representative of Gram-positive bacteria and *E.coli* as a representative of Gram-negative bacteria for the study of hydrogel (Figure 4). As shown in Figure 4A and C, in PTKH1, PTKH2 and PTKH3 groups, 89.7%, 92.6% and 95.3% of *E.coli* was eliminated as compared to control group. Similarly, colony number of *S.aureus* (Figure 4B and D) was reduced by 93.7%, 96.9%, and 98%, respectively. The phenolic hydroxyl group in TA offer multiple sites for hydrogen-bonding interactions with peptidoglycan (an important component of the bacterial cell wall), which helps to capture bacteria [47, 48].

### *In vitro* cell proliferation assay

**Figure 5.**
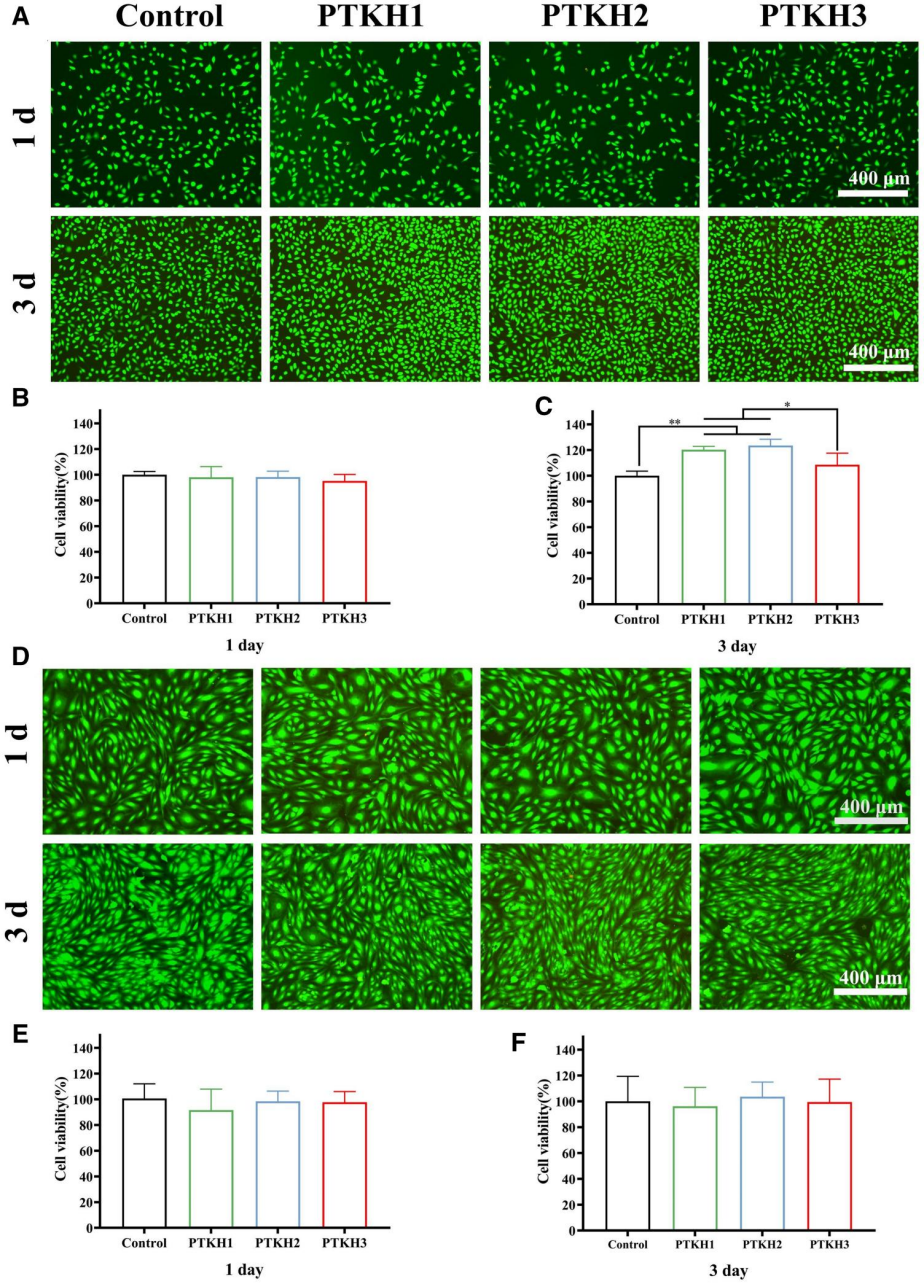
PTKH hydrogel cell proliferation assay. (**A**) Live/dead staining photos of L929 cells cultured with different hydrogel extract for 1 and 3 days; (**B**) The corresponding 1-day survival rate of L929 cells; (**C**) 3-day survival rate of L929 cells; (**D**) live/dead staining photos of HUVECs cells cultured with different hydrogel extract for 1 and 3 days; (**E**) 1-day survival rate HUVECs cells; (**F**) 3-day survival rate of HUVECs cells. *P < 0.05, **P < 0.01.

The *in vitro* cytotoxicity of PTKH hydrogel to L929 cells and HUVECs cells was investigated. The cell survival rate of L929 fibroblasts cultured with the PTKH extract for 1 day was higher than 95% (Figure 5A and B), there was no discernible difference between the control group and the experimental groups. The survival rate of L929 cells in the PTKH3 hydrogel group at 1 and 3 days showed a decreasing trend compared with that of the PTKH1, PTKH2 hydrogel group, which appeared due to the fact that too high concentration of TA would have a certain effect on the biocompatibility of cells. The 3 days culture experiment showed a significantly higher proliferation rate in PTKH1 and PTKH2 groups than the control group (Figure 5A–C). On the other hand, the HUVECs results revealed no significant difference between the experimental groups and the control group (Figure 5D and F). These results implied all three tested PTKH hydrogels are biocompatible and sensitivity of cells to hydrogel varied from each other.

### Silicate ions released from PTKH promoted cell migration

The acceleration of cell migration is crucial for wound repair. HUVECs are highly proliferative, capable of forming capillaries in angiogenesis. Numerous studies have shown that silicate ions at effective concentrations can stimulate the proliferation of HUVECs, up-regulating the expression of vascular endothelial growth factor (VEGF) [49], and ultimately stimulate pro-angiogenesis. Besides, silicate ions are found highly biologically active in terms of activation of extracellular vesicles of endothelial progenitor cells, as well as enhancement of paracellular secretory signal transduction [50].

**Figure 6.**
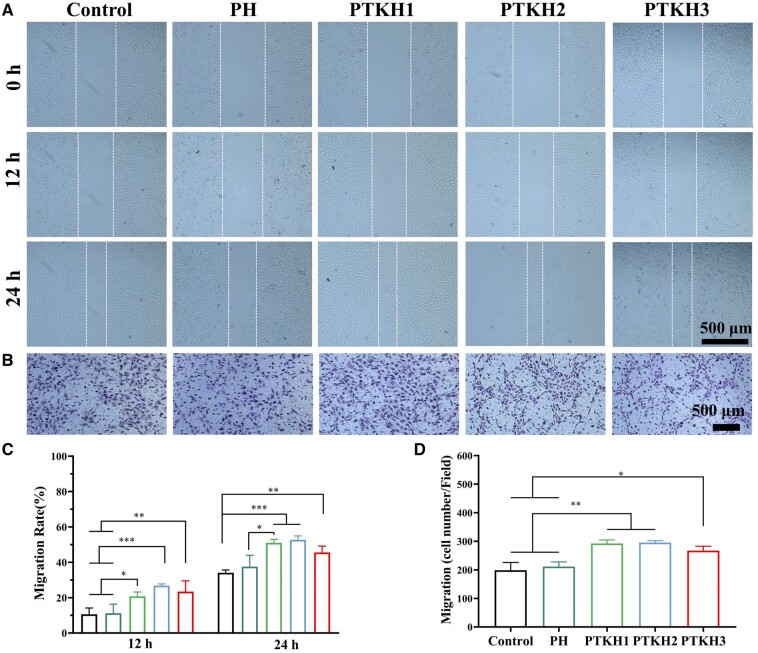
Effect of silicate ion release on the migration of HUVECs. (**A**) Representative microscopic images of HUVECs migration at different time points (0 h, 12 h, 24 h) in different test groups; (**B**) Crystal violet staining of transwell images of HUVECs migrated to the lower chamber in different treatment groups; (**C**) quantitative analysis of HUVECs migration during scratch testing; (**D**) quantitative analysis of HUVECs migration in transwell. *P < 0.05,**P < 0.01, ***P < 0.001.

Exploring the effect of different hydrogels on HUVECs migration, we used PH and PTKH hydrogels to assess the migration capability of cells by cell scratch assay and transwell assay. It is evident from Figure 6A and C that at 12 h, the control (10.6%) and PH (11.2%) groups had the least cell migration area compared to the PTKH1 (20.7%), PTKH2 (26.8%) and PTKH3 (23.4%) hydrogel groups. The results at 24 h showed that PTKH1 (51%), PTKH2 (52.7%) and PTKH3 (45.5%) hydrogel groups showed faster cell migration.

The PH, PTKH1, PTKH2 and PTKH3 hydrogels were also subjected to transwell experiments. As shown in Figure 6B and D, the results indicated that there was a limited difference in the number of cells recruited from the control and PH groups, whereas more HUVECs were recruited into the lower lumen from the PTKH1, PTKH2 and PTKH3 hydrogel groups.

These observation demonstrated the promoting effect of SiO**2-3** on cell migration and recruitment, which was further confirmed by quantitative analysis (Figure 6D). This is similar to previous reports that silicate ions promote the migration of endothelial cells by stimulating them [51, 52].

### *In vitro* tube forming and angiogenic differentiation

**Figure 7.**
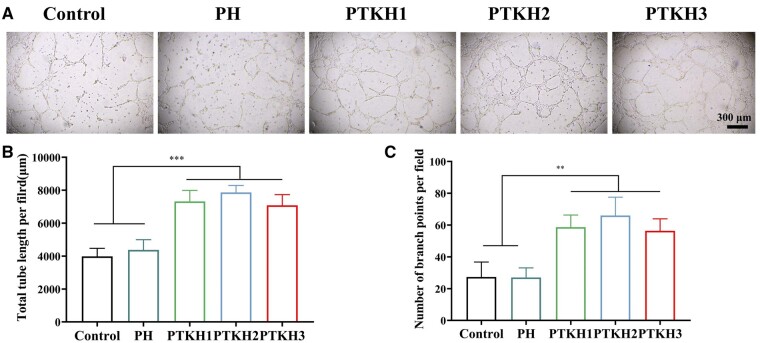
*In vitro* tube forming. (**A**) Microscopic images of tube formation by HUVECs on matrix gel; (**B**) length of HUVECs tube formation per field; (**C**) number of branch per field of HUVECs. **P < 0.01, ***P < 0.001.

We also investigated the *in vitro* tube-forming ability of HUVECs using extracts from hydrogels. As shown in Figure 7A, HUVECs treated with control and PH hydrogels formed loose and discontinuous tube networks after 6 h on Matrigel. In contrast, HUVECs treated with PTKH1, PTKH2 and PTKH3 hydrogels formed more complex and coherent tube networks. The length and number of branching points of the formed tubular networks were also quantified (Figure 7B and C) indicating that SiO**2-3** enhanced the *in vitro* tube-forming ability of HUVECs; we used qRT-PCR to analyze the expression of angiogenic gene VEGFA in HUVECs. The expression level of VEGFA in HUVECs from the PTKH1, PTKH2 and PTKH3 groups was significantly higher than that in the control and PH groups (Supplementary Figure S5).

According to literature, silicate ions are effective in promoting blood vessel formation mainly by promoting growth factors and thus regulating angiogenesis, such as VEGF and basic fibroblast growth factor (bFGF), as well as eNOS and HIF-1α included downstream angiogenic molecules [53–55].

### *In vivo* diabetic wound healing

**Figure 8.**
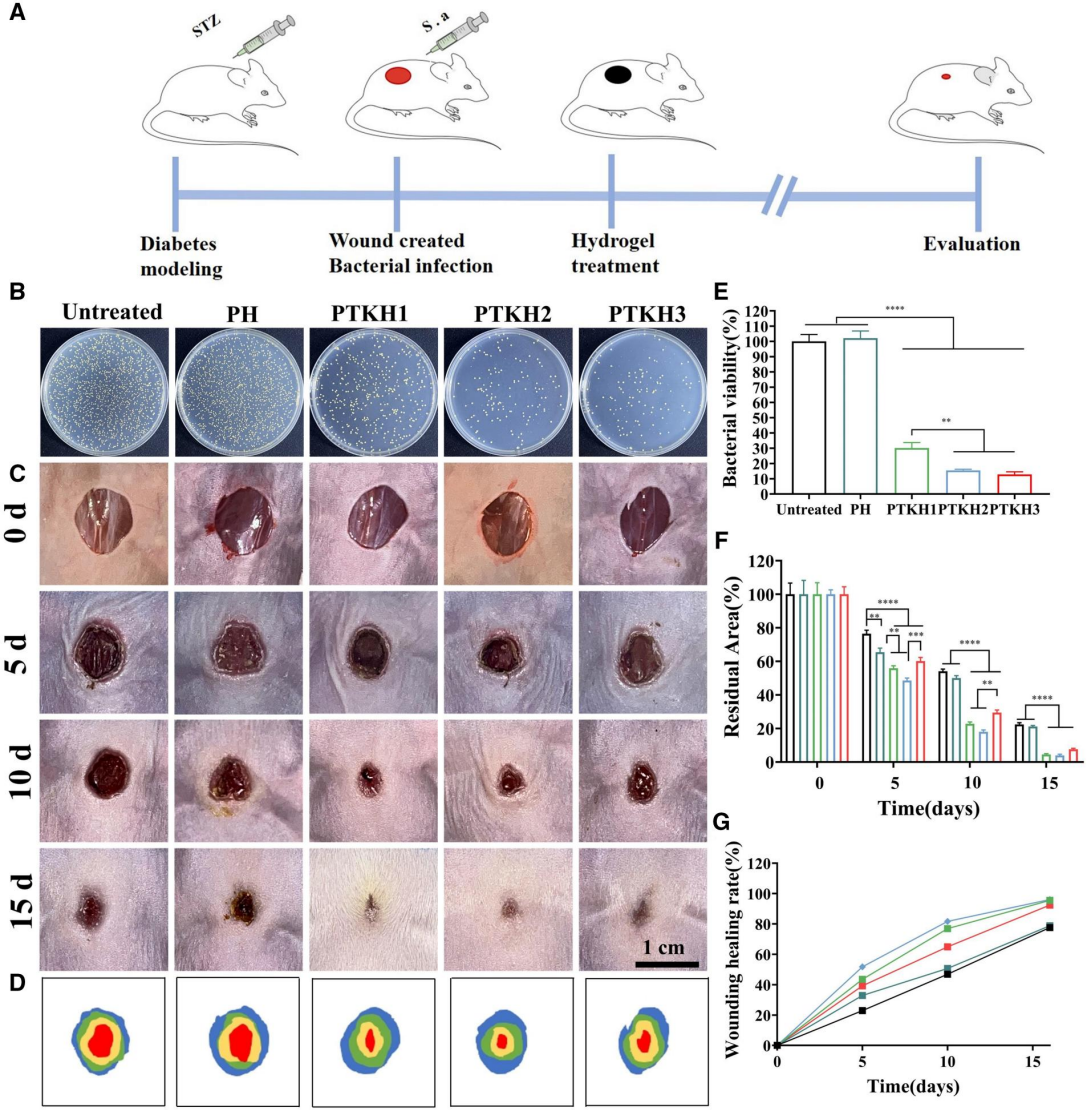
Wound healing at different points in time. (**A**) Schematic of animal experiment; (**B**) colony density of *S.aureus* harvest from skin wound after 3 days of treatment; (**C**) representative photos of wounds at different time intervals; (**D**) simulation of the wound healing process; (**E**) quantification of wound residual area; (**F**) quantification of wound healing rate; (**G**) quantitative analysis of *S.aureus* in different test groups. **P < 0.01, ***P < 0.001 and ****P < 0.0001.

PTKH hydrogels were further applied in *in vivo* wound healing using an *S.aureus-*infected diabetic BALB/c mouse [30] model (Figure 8A). After 3 days, the bacterial density in wound sites was evaluated via counting the colony-forming units (Figure 8B and E). As can be seen, 68.9%, 84.5% and 87.1% of *S.aureus* was eliminated in the PKTH1, PKTH2 and PKTH3 groups, while the untreated and PH groups showed similar colony density. The gradual change in wound region size was also photographed to evaluate the therapeutic efficiency of different hydrogel dressings (Figure 8C and D). As time proceeded, wound closure was witnessed in PTKH groups. As can be seen in Figure 8F, wounds in the PTKH2 group exhibited a reduction to 51.4% on Day 5, a value much higher than those observed in the untreated (23.6%), PH (34.5%), PTKH1 (44.1%), and PTKH3 groups (39.9%). At Day 10, the healing wound area of PTKH2 was found to be 82%, much higher than the untreated (45.9%), PH (49.9%), PTKH1 (77.2%) and PTKH3 groups (70.6%), which can also be witnessed in the simulation of the wound healing process. The crosslink density-induced swelling ability decrease as well as the high TA content may explain the difference between PTKH3 and other PTKH hydrogels. It was also found that healing outcome in the PH group was better than the untreated one, which is attributed to the exudate management and moist environment creation capabilities of PH in the absence of active agents.

**Figure 9.**
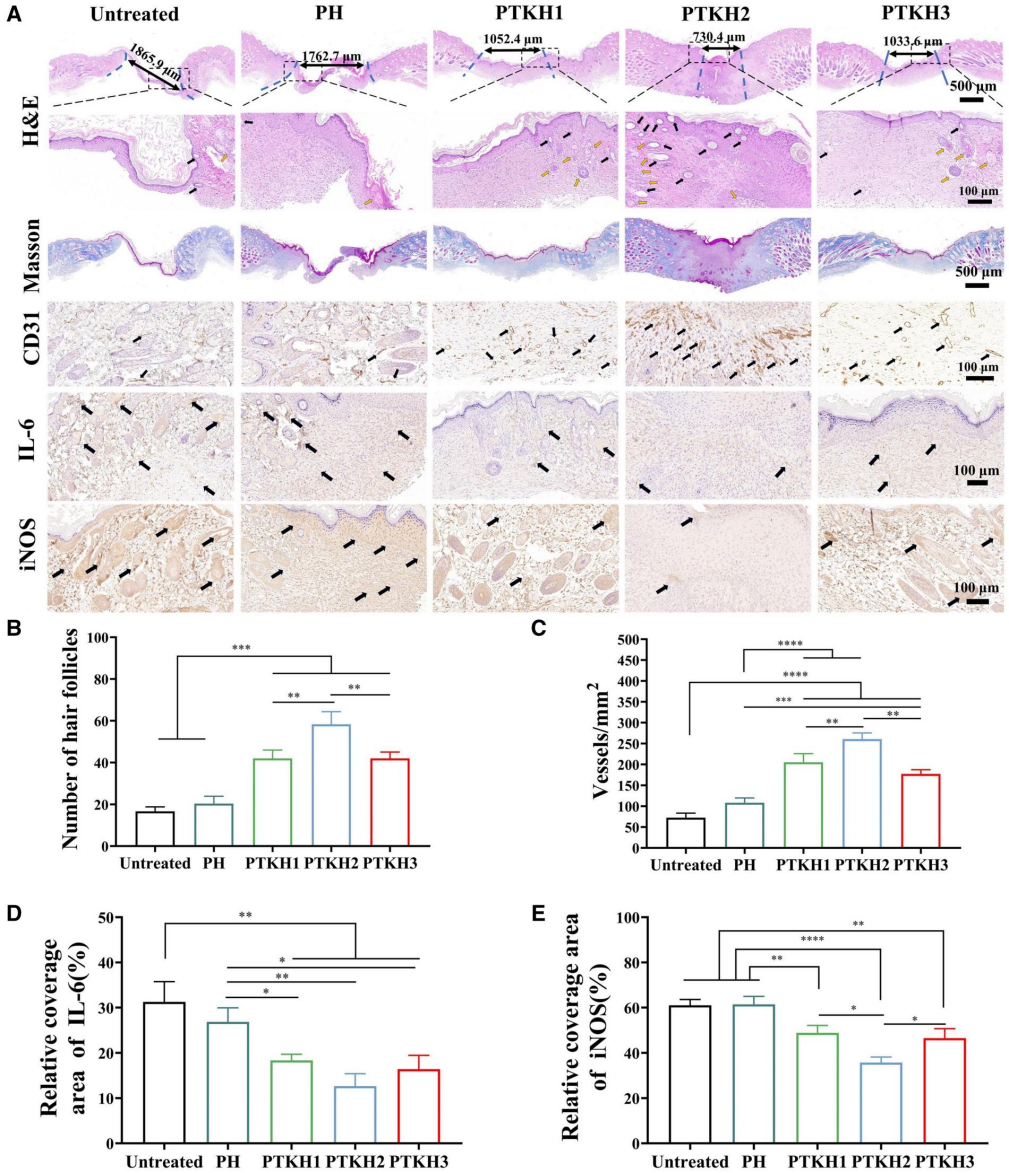
Histologic assessment of PTKH hydrogel for wound healing. (**A**) H&E staining images of wound tissue healing after 15 days with black arrow indicating hair follicles and yellow arrow indicating blood vessel, Masson’s trichrome staining results, CD31 immunohistochemical staining with black arrows indicating vascular specific markers, IL-6 immunohistochemical staining with black arrow indicating specific markers, iNOS immunohistochemical staining with black arrow indicating specific markers; (**B**) quantitative analysis of hair follicles; (**C**) quantitative analysis of vessel formation; (**D**) quantitative analysis of IL-6 and (**E**) quantitative analysis of iNOS. *P < 0.05, **P < 0.01, ***P < 0.001 and ****P < 0.0001.

To further assess the quality of neoplastic skin in wound repair, we performed hematoxylin and eosin (H&E) and Masson staining of regenerated wound skin tissue (Figure 9A). After 15 days of tissue healing, H&E tissue sections of wounds from diabetic-stained mice showed that the granulation tissue thicknesses of PTKH groups were significantly higher than the untreated control group. Similarly, the lengths of the wound were reduced in the order of untreated group (1.86 mm), PH hydrogel (1.76 mm), PTKH1 (1.05 mm), PTKH3 (1.03 mm) and PTKH2 (0.73 mm) as well. Likewise, Masson staining results showed that the results of PTKH and untreated groups had similar wound healing as H&E staining results. Among them, the PTKH2 group had the highest content and distribution of collagen fibers among all tested groups. The deposition of collagen is crucial in the process of wound healing, which also indicates that the PTKH2 group has faster wound healing.

Repair of the hair follicle is equally important in wound healing, serving to maintain the normal function of the skin. From H&E staining sections, it can be observed that the PTKH2 group had a higher number of hair follicles, while the PH group and the untreated group possessed fewer hair follicles, as quantified by the number of hair follicles in Figure 9D.

New blood vessel formation is acknowledged to be critical for diabetic wound healing. Consequently, we chose to use CD31 immunohistochemistry as a study of wound neovascularization. The HE staining and immunohistochemical CD31 staining (Figure 9A) demonstrated the superiority of PTKH over PH and untreated groups in vascular expression. Likewise, quantitative analysis of vessel formation shown in Figure 9C indicated that the blood vessel density of the PTKH2 group was significantly higher than that of the untreated and PH groups.

We chose to assess wound inflammation by IL-6 and iNOS immunohistochemistry. In the presence of PTKH, the IL-6 expression was greatly inhibited, with the expression greatly reduced from 31.3% of the untreated group and 25.9% of the PH hydrogel group to 18.3% of PTKH1, 16.1% of PTKH3 and 12.6% of PTKH2, and the expression of iNOS was also markedly inhibited (Figure 9A, D and E). This observation may be because TA and silicate exerted anti-inflammatory and anti-bacterial functions *in situ*.

All of the data show that after being treated by PTKH hydrogels, the wound microenvironment was reconstructed, simultaneously suppressing ROS and infection, promoting cell proliferation and collagen deposition, and augmenting hair follicle regeneration and angiogenesis. We provide a strategy of injectable self-curing with PVA as a dressing applied to the wound site, which can be combined with bioactive materials and growth factors in the future for more applications in soft tissue repair.

## Conclusions

In this study, a TA and silicate functionalized PVA–HA hydrogel PTKH was developed. Compared with the original hydrogel, the co-presence of TA and silicate enabled the sol–gel transition of the hydrogel *in situ* to generate an injectable and adhesive dressing for chronic diabetic wound healing acceleration. Through tailoring TA and silicate-induced crosslinking density, the mechanical, rheological and swelling properties of as-prepared hydrogel could be regulated. In spite of that, *in vitro* results demonstrated that this as-prepared ‘all-in-one’ hydrogel was biocompatible and blood compatible, capable of eliminating ROS generation, suppressing bacterial infection, promoting cell migration and angiogenesis. More importantly, *in vivo* model confirmed the feasibility of this hydrogel to promote skin wound healing and tissue regeneration by reducing inflammation and infection levels, accelerating collagen deposition, promoting blood vessel and hair follicle formation.

## Supplementary Material

rbae053_Supplementary_Data

## Data Availability

Data will be made available on request.
