# Supplementary material for: Tannic acid and silicate-functionalized polyvinyl alcohol–hyaluronic acid hydrogel for infected diabetic wound healing
Source: Regen Biomater. 2024 May 13;11:rbae053. doi: 10.1093/rb/rbae053 (PMC11176089; doi:10.1093/rb/rbae053)
Supplement: rbae053_Supplementary_Data [file rbae053_supplementary_data.docx]

**Supporting Information**

**Tannic acid and silicate functionalized polyvinyl alcohol-hyaluronic acid hydrogel for infected diabetic wound healing**

Zhentian Diao^a^,Longkang Li^a^ ,Huan Zhou^b,*^,Lei Yang^b,*^

*^a^ School of Materials Science and Engineering, Hebei University of Technology, Tianjin, 300131, China*

*^b^ Center for Health Science and Engineering, Hebei Key Laboratory of Biomaterials and Smart Theranostics, Tianjin, 300131, China*

** Corresponding author. E-mail addresses*: zhouhuan@hebut.edu.cn (H. Zhou), ylei@hebut.edu.cn (L. Yang) .

**1. Experimental Methods**

**1.1 PTKH hydrogel injection behavioral test**

The pre-crosslinked PTKH hydrogels were added into 20 mL syringes, and the push-injection force of PTKH hydrogels was investigated by using a 21 G needle. The syringes filled with different PTKH hydrogels were transferred to a mechanical testing machine, and the compression speed was set to 10 mm/min to analyze the push-injection force-displacement curves of the corresponding PTKH hydrogels.

**1.2 Adhesion performance test**

The adhesion strength of PTKH hydrogel on the corresponding substrate material was tested using a mechanical testing machine. First, the corresponding substrate material was fixed in the upper and lower fixtures of the mechanical testing machine and the PTKH hydrogel was placed on the surface of the substrate material in the lower fixture. Subsequently, the upper fixture was moved downward and the PTKH hydrogel was compressed with a pressure of 2 N. The PTKH hydrogel was placed on the surface of the substrate material. When the pressure was relaxed to 0 N, the PTKH hydrogel was stretched at 20 mm/min until it left the substrate material and the maximum stress was recorded and defined as the adhesion strength in terms of its ratio to the contact area.

**1.2 qRT-PCR test**

The expression level of VEGF-A was investigated by qRT-PCR. RNA was extracted using an extraction kit (RNAeasy™ Animal RNA Isolation Kit Spin Column, Beyotime, China) according to the manufacturer's test protocol. and RNA was converted to cDNA, and RT-PCR quantification was performed by a real-time fluorescence quantitative PCR detector (CFX Connect™). Glyceraldehyde-3-phosphate dehydrogenase (GAPDH) was used as an endogenous control and calculated using 2 ^- ΔΔCT^. The primers are detailed in Table S1.

Table S1. Primer sequences for RT-qPCR.

| Primer | Forward | Reverse |
| --- | --- | --- |
| GAPDH | CTCTTCAGTTCGTGTGTGGAGAC | CAGCCTCCTTAGATCACAGCTC |
| VEGF-A | TTCCTGTAGACACACCCACC | CAGGGATTTTTCTTGTCTTGCT |

**1.3 ICP test**

PTKH1, PTKH2, and PTKH3 were immersed in PBS buffer (50 mg/mL), and Si^4+^ was measured by ICP-OES at 12, 24, and 72 h, respectively.

**1.4 PTKH hydrogel solution UV-Vis test**

PTKH1, PTKH2 and PTKH3 hydrogels were dissolved in PBS buffer (50 mg/mL) and the samples were removed and filtered at 12, 24 and 72 h. Subsequently, the PBS buffer was used for calibration, and then the PTKH soaking solution at each time point was diluted by a certain number of times and UV-visible spectral curves were performed in the wavelength range of 200-500 nm.

**2. Supporting Figures**


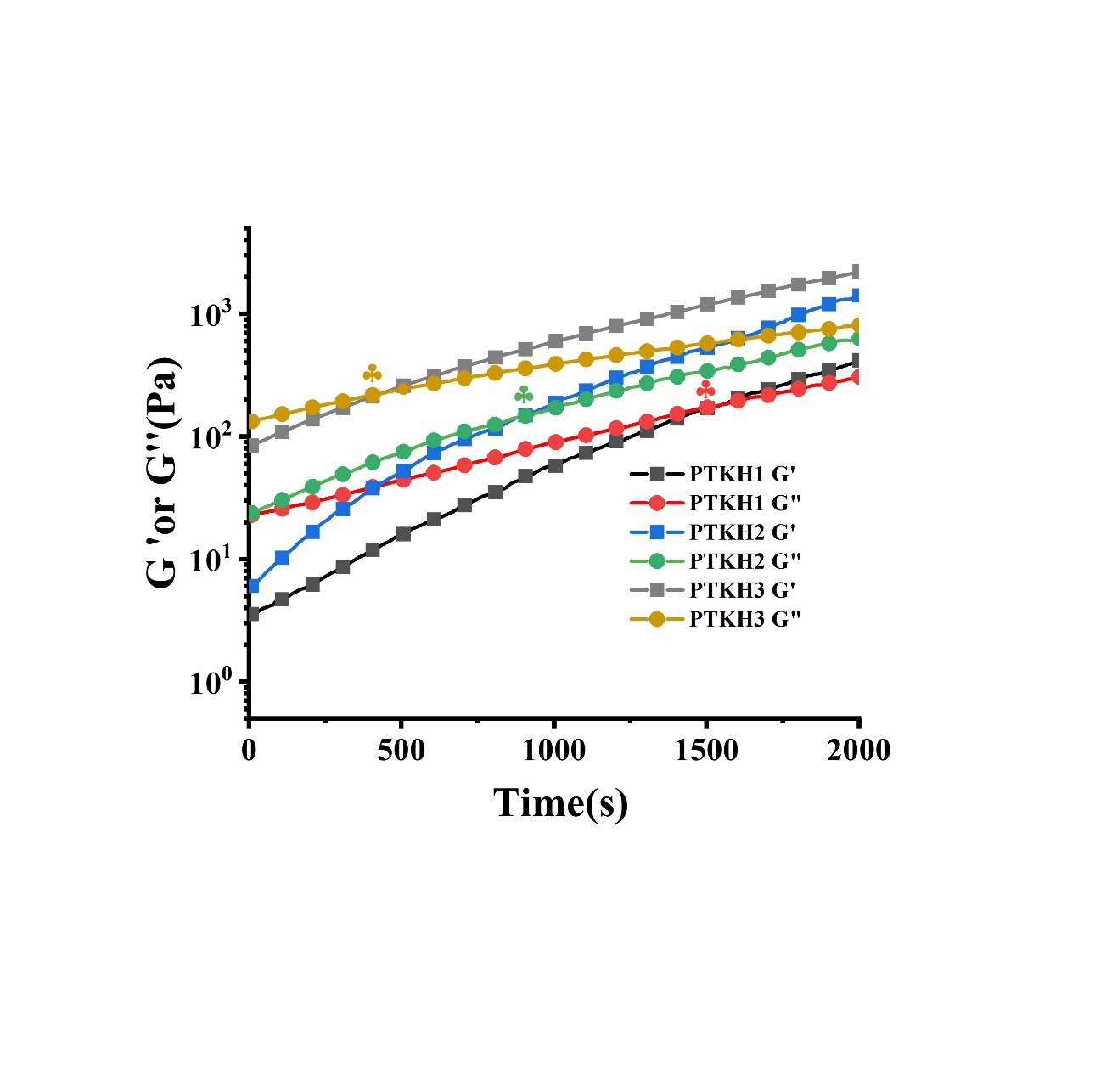


**Figure S1.** Rheogram of gelation time for different of PTKH.


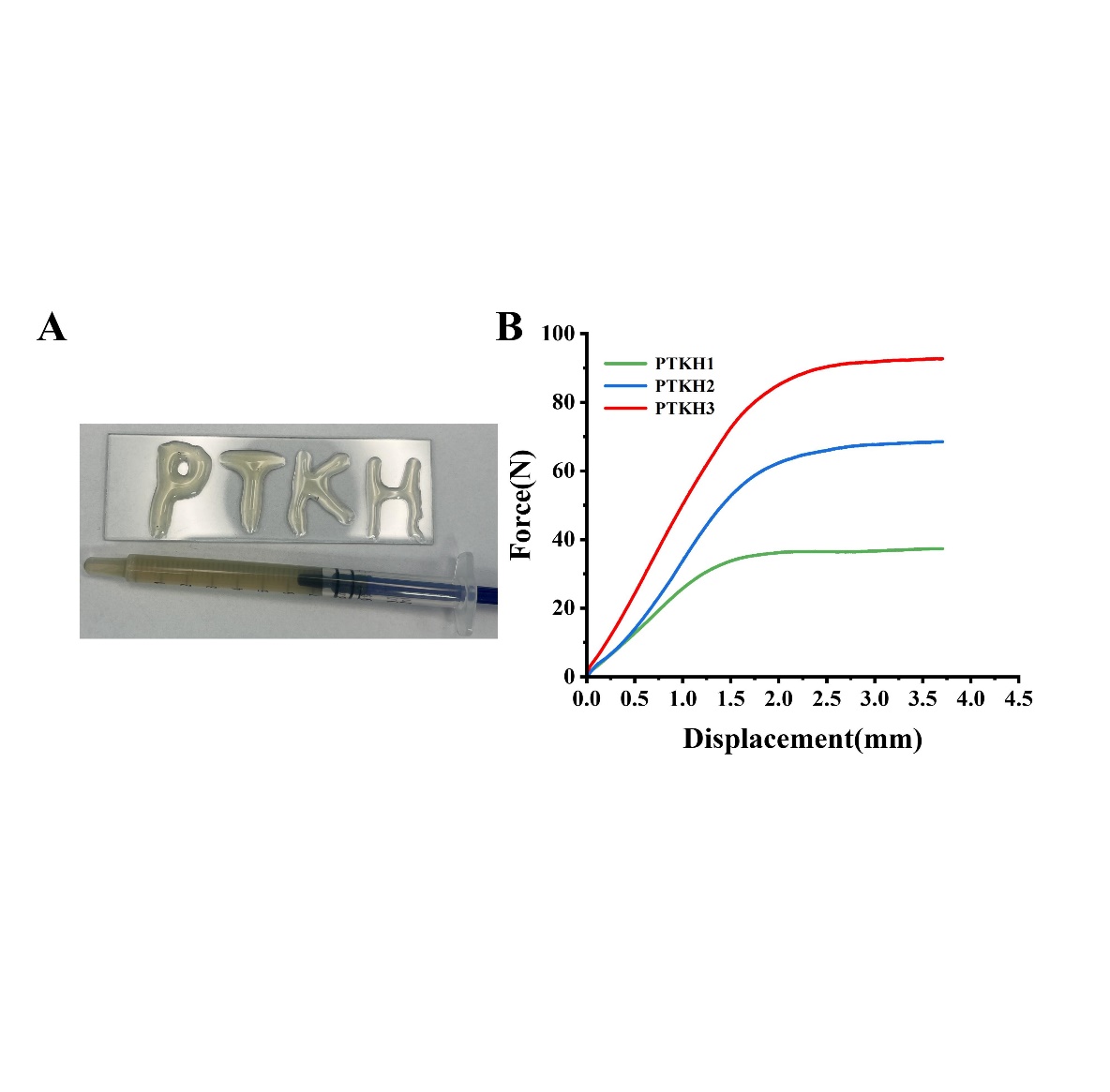


**Figure S2.** (A) Schematic diagram of uncured PTKH injectable hydrogel injection; (B) Push-injection force-displacement curve of uncured PTKH injectable hydrogel.


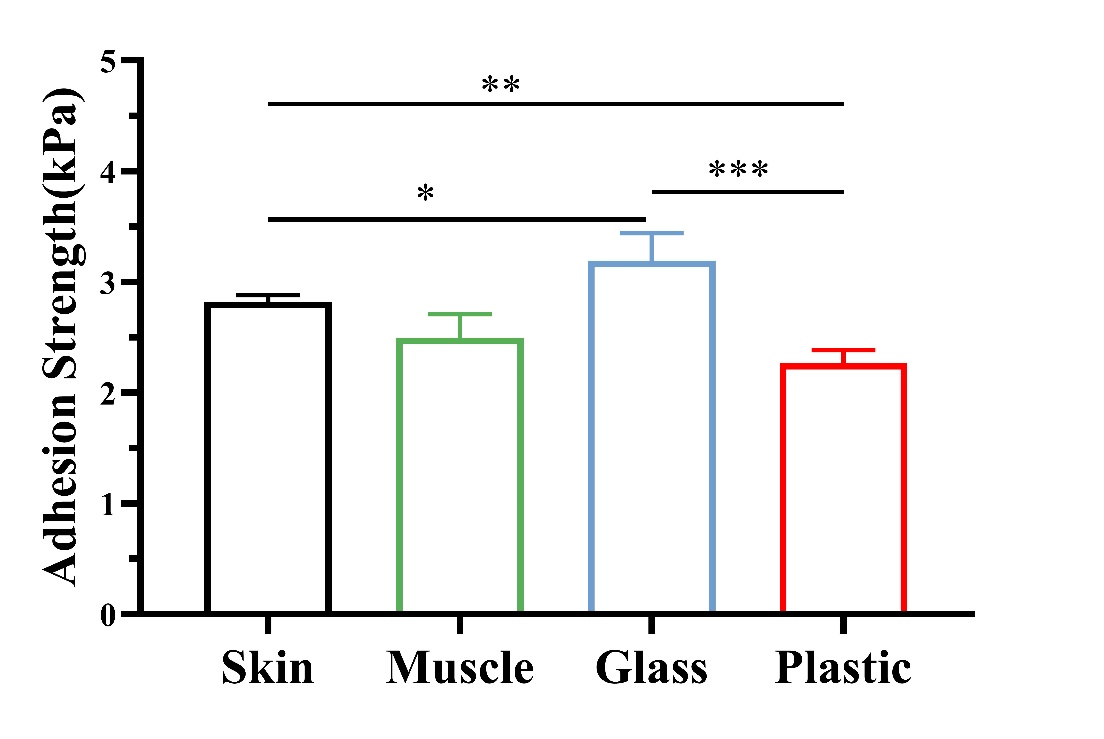


**Figure S3.** Adhesion ability of PTKH hydrogel on different material surfaces. *p<0.05, **p<0.01, ***p<0.001 and ****p<0.0001.


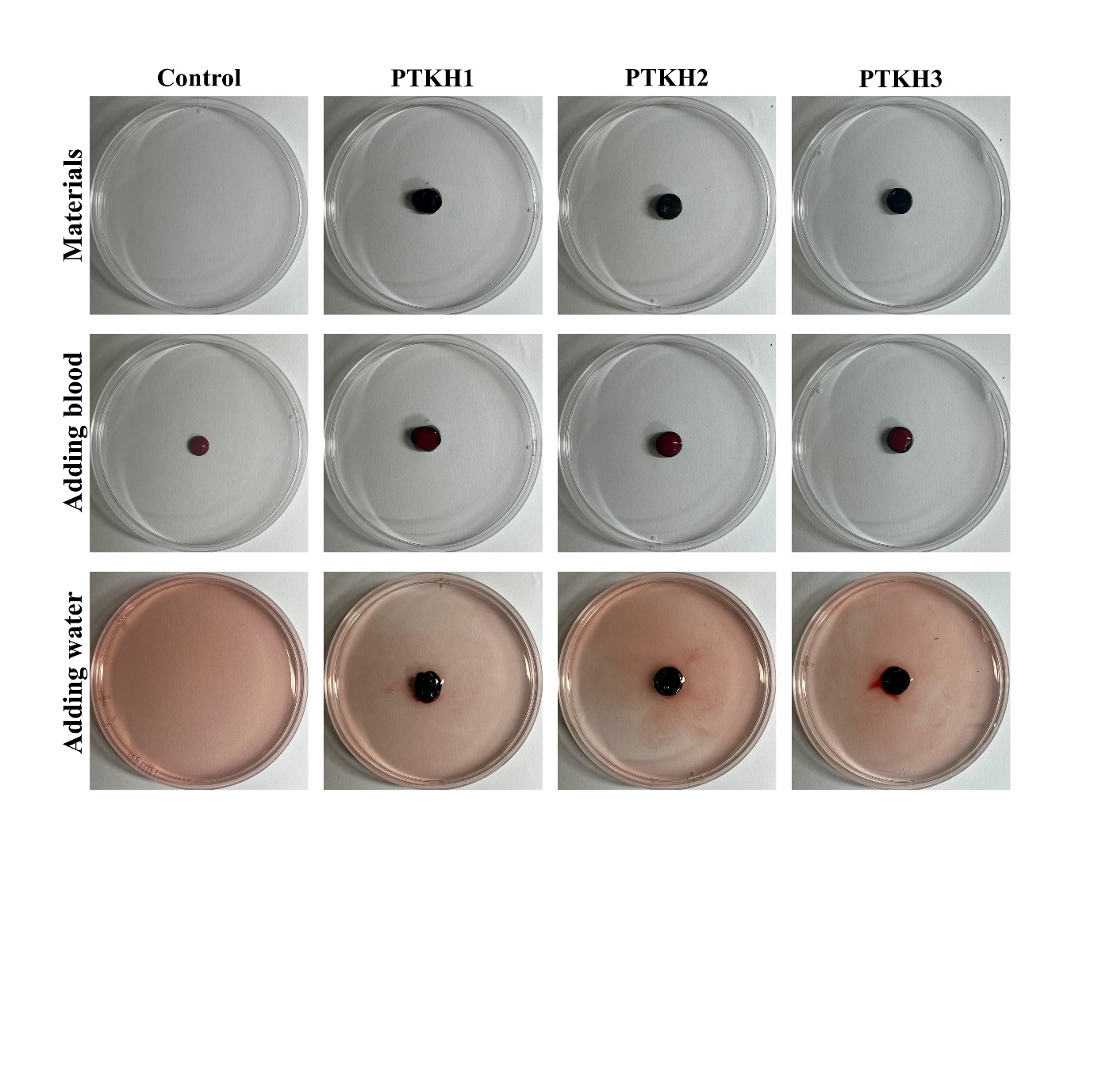


**Figure S4.** Photos of coagulation process of different hydrogels.


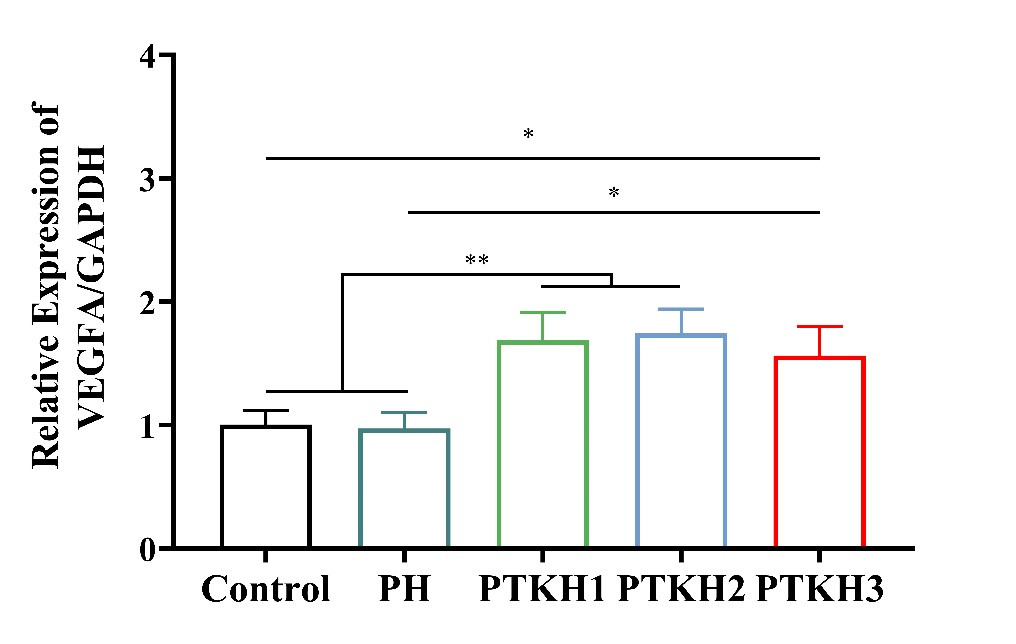


Figure **S5**. Relative expression of VEGFA in HUVECs treated in different groups. *p<0.05, **p<0.01, ***p<0.001 and ****p<0.0001.


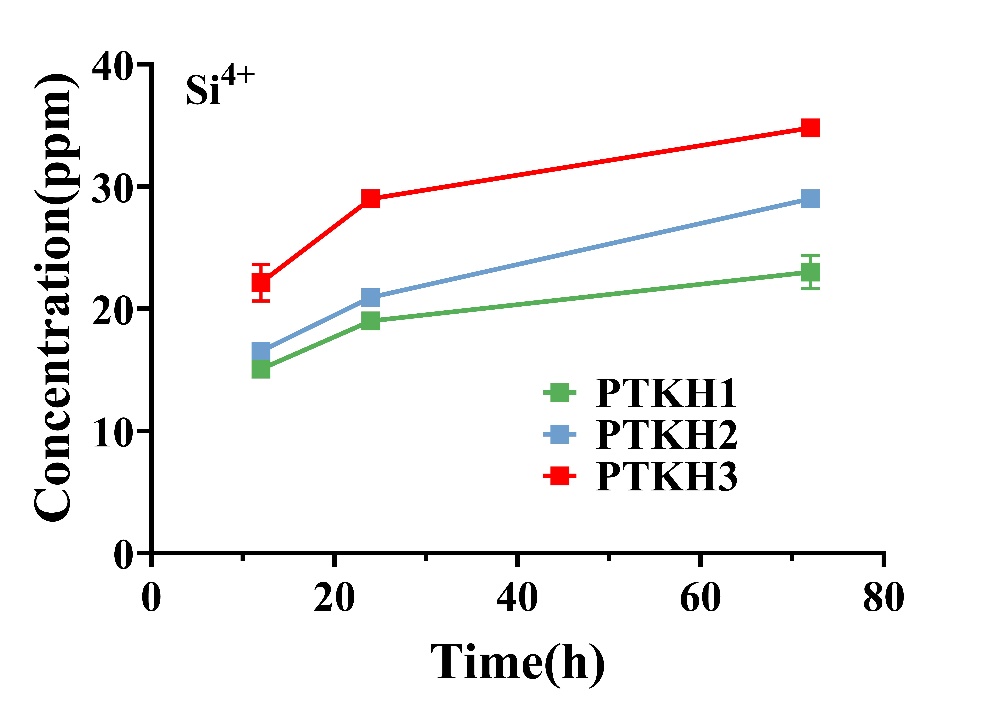


**Figure S6.**Si^4+^ ion release profiles of PTKH hydrogels in PBS buffer at different time points.


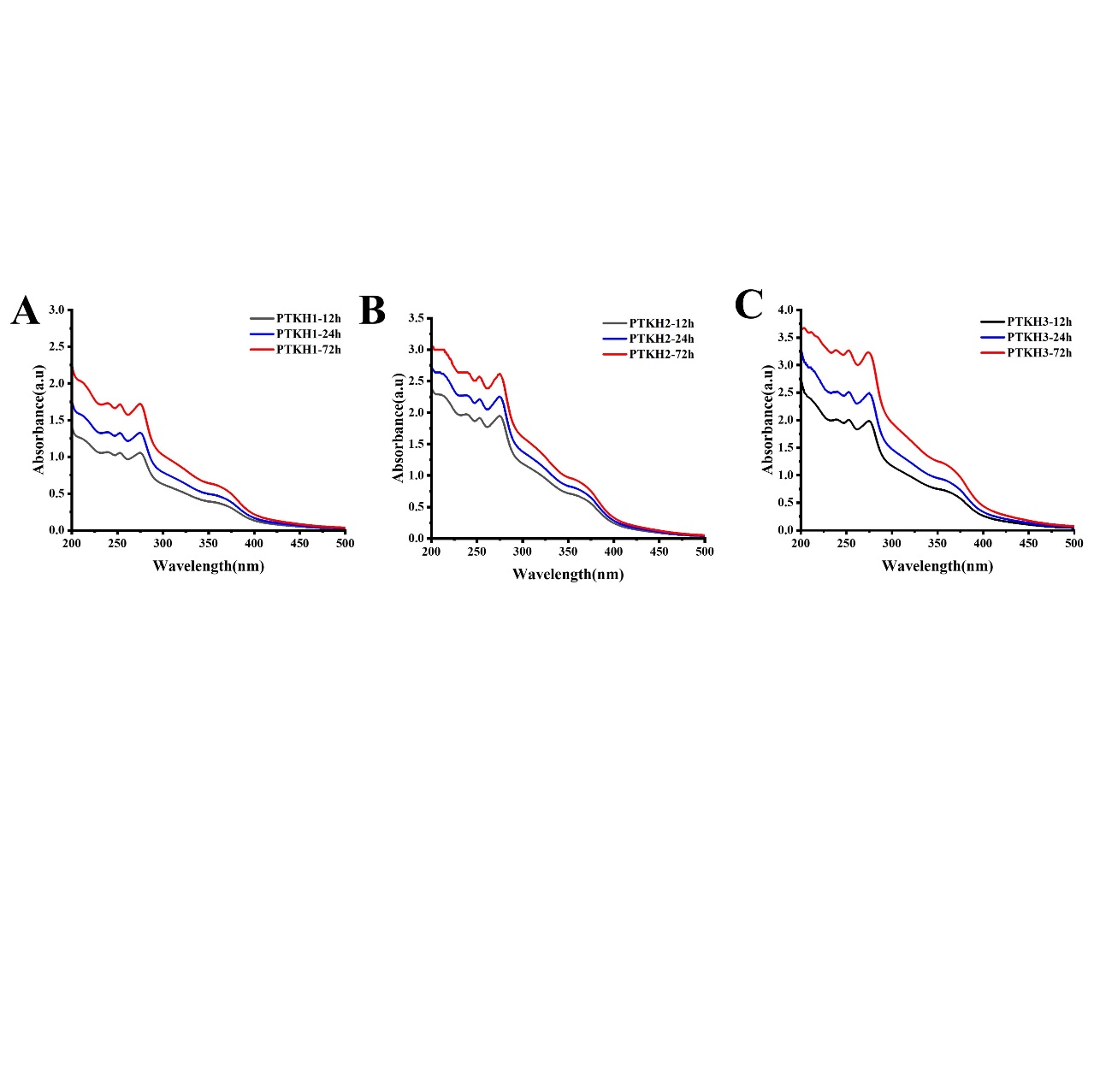


**Figure S7.** UV-Vis spectra of PBS solutions of PTKH hydrogels at 12, 24, and 72 h.
